# Prognostic value of *TP53* expression and *MGMT* methylation in glioblastoma patients treated with temozolomide combined with other chemotherapies

**DOI:** 10.1007/s11060-021-03723-9

**Published:** 2021-03-04

**Authors:** Maher Kurdi, Nadeem Shafique Butt, Saleh Baeesa, Badrah Alghamdi, Yazid Maghrabi, Anas Bardeesi, Rothaina Saeedi, Ashraf Dallol, Fawaz Mohamed, Mohammed O. Bari, Alaa Samkari, Ahmed I. Lary, Shadi Alkhayyat

**Affiliations:** 1 Department of Pathology, Faculty of Medicine in Rabigh, King Abdulaziz University, Jeddah, Kingdom of Saudi Arabia; 2 Department of Family and Community Medicine, Faculty of Medicine in Rabigh, King Abdulaziz University, Jeddah, Kingdom of Saudi Arabia; 3 Division of Neurosurgery, Faculty of Medicine, King Abdulaziz University, Jeddah, Kingdom of Saudi Arabia; 4 Department of Physiology, Faculty of Medicine, King Abdulaziz University, Jeddah, Kingdom of Saudi Arabia; 5 Department of Neuroscience, King Faisal Specialist Hospital and Research Center, Jeddah, Kingdom of Saudi Arabia; 6 Center of Excellence in Genomic Medicine Research, Faculty of Applied Medical Science, King Abdulaziz University, Jeddah, Kingdom of Saudi Arabia; 7 Department of Pathology, King Abdulaziz University Hospital, Jeddah, Kingdom of Saudi Arabia; 8 Department of Pathology and Laboratory Medicine, King Saud Bin Abdulaziz University for Health Science, Jeddah, Kingdom of Saudi Arabia; 9 Section of Neurosurgery, Department of Surgery, King Abdulaziz Medical City, Jeddah, Kingdom of Saudi Arabia; 10 Division of Oncology, Faculty of Medicine, King Abdulaziz University, Jeddah, Kingdom of Saudi Arabia

**Keywords:** Glioblastoma, *MGMT* promotor methylation, *TP53* mutation, Temozolomide

## Abstract

**Objective:**

To assess the recurrence interval and predictive significance of *TP53* expression and O6-methylguanine-DNA methyltransferase (*MGMT*) promoter methylation in glioblastomas treated with radiotherapy and combined chemotherapies, including temozolomide, lomustine, procarbazine and bevacizumab.

**Method:**

We reviewed the clinical outcomes of 52 totally resected glioblastoma patients, who received conventional radiotherapy and temozolomide with other chemotherapeutic agents. Correlation of *TP53* expression and *MGMT* promotor methylation with recurrence interval was analyzed using Kaplan Meier estimates.

**Results:**

No significant association was found between *MGMT* promotor methylation and *TP53* expression in glioblastomas (*P*-value = 0.158). Patients with non-methylated *MGMT* who received temozolomide chemotherapy with other chemotherapeutic agents showed significantly later recurrence (*P*-value = 0.007) compared with patients with non-methylated *MGMT* who received temozolomide alone. No significant difference was found in recurrence interval among glioblastoma patients with methylated *MGMT* who received temozolomide alone or with other chemotherapies (*P*-value = 0.667). Moreover, patients with non-*TP53*-expressing tumors who received temozolomide with other chemotherapies had significantly later recurrence (*P*-value = 0.04) compared with patients who received temozolomide alone.

**Conclusion:**

Totally resected glioblastoma patients, with non-methylated *MGMT* or non-*TP53*-expressing tumors treated with radiotherapy and combined chemotherapies had a reduced chance of tumor recurrence and a more favorable outcome. Furthermore, both *MGMT* and *TP53* are independent prognostic factors for glioblastoma.

## Introduction

Glioblastoma is the most common primary brain tumor in adults. The standard treatment includes surgical resection followed by radiotherapy (RT) and chemotherapy, mostly with the alkylating agent, temozolomide (TMZ). Despite these regimens, outcomes remain poor [1]. Although RT with TMZ is the first option for newly diagnosed glioblastoma patients, chemotherapy resistance is common. Several parameters are used to predict the prognosis of patients with glioblastoma, including methylation of the O6-methylguanine-DNA methyltransferase gene (*MGMT*) and mutation of the tumor suppressor gene, Tumor Protein 53 (*TP53*) [2, 3]. The regulation of *MGMT* is complex and involves *TP53*, which is required for induction and also for down-regulation to basal levels [4]. Although *MGMT* methylation is more frequent in tumors expressing mutant *TP53*, the association between these two factors is uncertain. Hence, high levels of *MGMT* activity with low *TP53* expression are associated with a high TMZ resistance rate. Not all glioblastoma patients with *MGMT* promoter methylation respond to alkylating agents, and even those who respond eventually experience relapse [5, 6]. *MGMT* promoter methylation is associated with improved overall survival in patients treated with RT plus temozolomide but not in patients initially treated with RT alone. Indeed, tumor progression-free survival of glioblastoma patients with *MGMT* methylation is better than that of patients with non-methylated glioblastoma. These findings do not correlate with *TP53* expression or *TP53* mutation status. Different strategies have been applied to overcome MGMT-mediated chemoresistance but none of them have significantly improved tumor progression-free survival.

Here, we tested 52 totally resected glioblastoma patients for *MGMT*-methylation and *TP53* expression. We then assessed the relationship between *MGMT* promoter methylation and *TP53* mutation with recurrence status in glioblastoma patients receiving single or combined chemotherapies. This is the first study of this association in Saudi Arabia.

## Material and methods

### Patient stratification

The study included 52 patients with primary glioblastomas who underwent total surgical resection at different medical institutions in Saudi Arabia between 2013 and 2019. The study was approved by the National biomedical Ethics Committee of King Abdulaziz University (HA-02-J-008). Histological diagnoses were made according to the World Health Organization (WHO) classification 2016 of CNS tumours. Clinical data, including patients’ age at diagnosis, sex, post-operative radiotherapy and chemotherapies, were taken from hospital reports. None of the patients had received preoperative radiation or chemotherapy. Results of *MGMT* promotor methylation were available for 36 patients from hospital records. The remaining 16 patients involved in this study underwent *MGMT* methylation status testing using tissue blocks. In total, 54 patients received a standard protocol of radiotherapy (RT) and chemotherapy after surgery while four patients received only radiotherapy. Three patients did not receive any kind of treatment after surgery because of poor health status. All patients who received chemoradiotherapy were stratified into two groups: Group (a) received temozolomide (TMZ) alone (n = 29), and Group (b) received TMZ plus other chemotherapeutic agents (n = 16). The additional chemotherapeutic agents were a chloroethylating agent (lomustine) with or without procarbazine and bevacizumab. Standard RT was given as a total dose of 60 Gy and the TMZ was given at 150–200 mg/m^2^ for 5 days every 28 days for 6–12 cycles. All patients enrolled in this analysis have unfortunately died.

### Tumor samples

Archival routinely formalin-fixed and paraffin-embedded (FFPE) tumor tissues were collected from 52 patients of Arabic descent, who were histologically diagnosed with glioblastoma. Hematoxylin and eosin (H&E)-stained sections were re-examined by a certified neuropathologist (MK) to ensure that the histopathological diagnosis was made based on the WHO’s classification. One unstained positive-charged slide from each of the 52 FFPE tissue blocks was prepared for TP53 immunostaining. Five 6-µ FFPE sections were obtained from each sample for *MGMT* methylation sequencing.

### Immunohistochemistry (IHC) for TP53

An anti-TP53 antibody was used to qualitatively identify wild-type and mutant TP53 in FFPE sections using an automated slide stainer. The IHC assay using a mouse monoclonal antibody (IgG1, kappa), directed against human TP53 (DO-7), was performed with the ultraView DAB detection Kit from Ventana on a BenchMark XT automated staining system. A protocol was established so that the entire assay procedure consisted of deparaffinization with EZ Prep at 75 °C, heat pre-treatment in Cell Conditioning medium (Ag unmasking) (CC1; Ventana) for 60 min and then primary incubation for 16 min at 37 °C. The slides were counterstained with Hematoxylin II for 16 min and bluing reagent was used for 16 min. After that, the slides were removed from the slide stainer and then immersed into successive alcohol buffers at different concentrations for 3 min. Sections in which > 10% of tumor cells were positively stained were defined as “Expressed TP53” and thus mutated (TP53-mt).

### DNA extraction and MGMT methylation sequencing

Thirty-six samples had been previously tested for *MGMT* methylation using a pyrosequencing method and the results were obtained from hospital records. For the remaining 16 samples, we used qualitative methylation-specific PCR (MSP) to detect *MGMT* methylation status. The MSP assay detects CpG island methylation with high sensitivity and specificity. Samples in which a methylated sequence was amplified were scored as methylation positive. The percentage of methylated amplicons detected in an unmethylated control was defined as the cut-off value to separate unmethylated from methylated glioblastomas.

H&E-stained sections from 16 FFPE tissue blocks were examined by a neuropathologist (MK) to select regions from which DNA could be extracted. DNA was isolated by standard procedures from selected tissue fragments containing a high percentage of tumor cells. DNA extraction was performed using the QIAamp DNA FFPE tissue kit according to the manufacturer’s instructions. DNA quantity and quality were determined using a NanoDrop spectrophotometer at A260/A280 and A260/A230. The concentration of DNA samples was normalized to 50 ng and bisulfite-converted using the EpiTect Bisulfite Kit (Qiagen) according to the manufacturer’s instructions. Qualitative detection of *MGMT* methylation was performed using MSP as described previously with modifications [7]. The forward and reverse primers targeting methylated and unmethylated exon 1 of the human *MGMT* gene are listed in Table 1 and correspond to those described by Esteller et al. [7]. The PCR Kit used was HotStarTaq plus DNA polymerase (Qiagen). Thermal cycling on a Veriti thermal cycler (Thermo Fisher) included an initial step at 95 °C for 2 min followed by 40 cycles of 30 s at 94 °C, 30 s at 52 °C, and 30 s at 72 °C for 10 min. In vitro methylated and non-methylated control DNA (Qiagen) was used in every run. The PCR products were visualized on 8% non-denaturing polyacrylamide gels and stained with ethidium bromide. Samples having only methylated PCR products and samples having both methylated and non-methylated PCR products were both scored as methylation positive.

**Table 1:** Primers for methylation-specific polymerase chain reaction (MSP) used for testing *MGMT* methylation

Primer	Sequence
MSP-MGMT-MetF	5′-TTTCGACGTTCGTAGGTTTTCGC-3′
MSP-MGMT-MetR	5′-GCACTCTTCCGAAAACGAAACG-3′
MSP-MGMT-UnMetF	5′-TTTGTGTTTTGATGTTTGTAGGTTTTTGT-3′
MSP-MGMT-UnMetR	5′-AACTCCACACTCTTCCAAAAACAAAACA-3′

### Statistical methods

Data are described as frequencies and percentages. Recurrence interval (RI) was calculated from the time of starting adjuvant therapy after surgical resection until the first day of tumor recurrence. Chi-Square, Fisher’s Exact and Mantel–Haenszel Chi-Square tests were used to explore the association of recurrence status, *MGMT* promotor methylation status, and *TP53* expression with various study factors. Kaplan Meier curves and log-rank tests were used to compare the distribution of recurrence time. All statistical analyses were performed using IBM SPSS1 ver. 24 statistical software (IBM Corp., Armonk, NY).

## Results

### Descriptive analysis

**Fig. 1:**
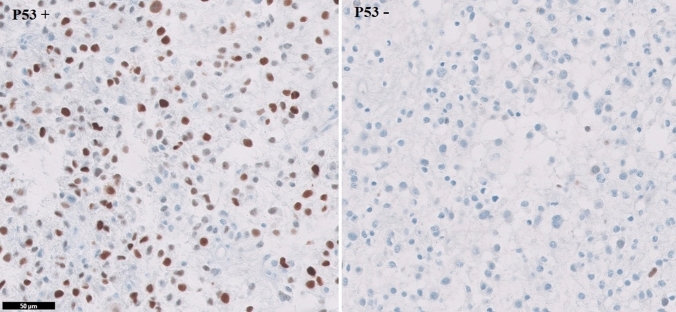
TP53 expression in glioblastoma; **a** positive expression of a *TP53* mutant; **b** negative expression of wild-type *TP53*. Scale bar 50 µm

Of the 52 enrolled patients, 21 were less than 50 years old, and 31 were aged 50 or over. All patients were with Arabic descent. The predominant tumor location was the frontal lobe (n = 20, 38.5%) followed by temporal and parietal areas (n = 28). Rare locations included the cerebellum and occipital lobe. There were equal numbers of glioblastoma cases with *MGMT*-methylation (n = 26) and without *MGMT* methylation (n = 26). Thirty-one tumors were positive for *P53* expression (59%) and 21 tumors lacked *TP53* expression (Fig. 1). Tumor recurrence occurred in 44% of patients within 1 year and in 55.8% of patients after more than 1 year. Table 2 summarizes these descriptive data.

**Table 2:** Distribution of descriptive data

	Number of patients (n = 52)
Age at diagnosis	
< 50 years	21.0 (40.4%)
≥ 50 years	31.0 (59.6%)
Sex	
Male	31.0 (59.6%)
Female	21.0 (40.4%)
Tumor location	
Frontal	20.0 (38.5%)
Temporal	14.0 (26.9%)
Parietal	14.0 (26.9%)
Occipital	2.0 (3.8%)
Cerebellar	2.0 (3.8%)
*MGMT* methylation profile	
Non-methylated	26.0 (50.0%)
Methylated	26.0 (50.0%)
*TP53* expression	
Negative	21.0 (40.4%)
Positive	31.0 (59.6%)
Adjuvant treatment	
None	3.0 (5.8%)
Radiation	4.0 (7.7%)
Radiation and chemotherapy	45.0 (86.5%)
Recurrence interval	
Before 1 year	23.0 (44.2%)
After 1 year	29.0 (55.8%)

### Statistical analysis

#### Correlation of age and MGMT methylation with recurrence interval

Glioblastomas with methylated *MGMT* were observed more frequently in patients older than 50; however, no significant difference in recurrence interval was observed between methylated and non-methylated *MGMT* cases in either age group (*P*-value = 0.670, *P*-value = 0.667) (Table 3).

**Table 3:** Age distribution of glioblastoma patients with *MGMT* methylation status

		Recurrence interval				P-value	Adjusted P-value
		Before 1 year		After 1 year			
		n	(%)	n	(%)		
Age at diagnosis	*MGMT* status						
< 50 Years	Non-methylated	7	(58.3)	5	(41.7)	0.670^b^	0.658^c^
	Methylated	4	(44.4)	5	(55.6)		
≥ 50 Years	Non-methylated	6	(42.9)	8	(57.1)	0.667^a^	
	Methylated	6	(35.3)	11	(64.7)		

No significant difference in recurrence rate was observed between patients with methylated and non-methylated *MGMT* in either age group

^a^Chi-Square Test; ^b^Fisher’s Exact Test; ^c^Mantel-Haenszel Chi-Square Test

#### Methylation analysis of the MGMT promoter and TP53 expression

Fifty-two tumor samples were available for *MGMT* promoter methylation and *TP53* expression analysis. Regardless of location, *MGMT* promoter methylation was identified in 18 cases with *TP53*-positive tumors (58%), while 13 *TP53*-positive cases had non-methylated *MGMT* tumors (Table 4). No significant association was found between *MGMT* promotor methylation and *TP53* expression (*P*-value = 0.158).

**Table 4:** Relationship between *MGMT* promotor methylation status and *TP53* expression

	MGMT status				P-value
	Non-methylated		Methylated		
	n	(%)	n	(%)	
*TP53* expression					
Negative	13	(61.9)	8	(38.1)	0.158^a^
Positive	13	(41.9)	18	(58.1)	

No significant association was found between *MGMT* promotor methylation and *TP53* expression

^a^Chi-Square Test

#### TP53 expression with different treatment modalities and recurrence interval

Although 88.9% of *TP53*-positive patients who received TMZ plus other chemotherapies had no tumor recurrence for over 1 year, no significant difference was found in recurrence rate with *TP53*-positive patients who received TMZ alone. Interestingly, around 86% of patients who were negative for *TP53* expression and who received TMZ plus additional chemotherapies did not have a recurrence within 1 year (*P*-value = 0.040). However, a significant difference in recurrence interval was observed between *TP53*-positive patients versus *TP53*-negtive patients who received different chemotherapeutic agents (adjusted *P*-value = 0.035) (Table 5).

**Table 5:** Analysis of *MGMT* methylation and *TP53* expression status versus recurrence rate in glioblastoma patients receiving different chemotherapies

	Recurrence interval				P-value	Adjusted P-value
	Before 1 year		After 1 year			
	n	(%)	n	(%)		
*MGMT* status						
Non-methylated						
Temozolomide	8	(61.5)	5	(38.5)	0.007^b^	0.041^c^
Temozolomide + other	0	(0.0)	8	(100.0)		
Methylated						
Temozolomide	6	(37.5)	10	(62.5)	0.667^b^	
Temozolomide + other	2	(25.0)	6	(75.0)		
*TP53* expression						
Negative						
Temozolomide	7	(63.6)	4	(36.4)	0.040^a^	0.035^c^
Temozolomide + other	1	(14.3)	6	(85.7)		
Positive						
Temozolomide	7	(38.9)	11	(61.1)	0.201^b^	
Temozolomide + other	1	(11.1)	8	(88.9)		

All patients with non-methylated *MGMT* who received temozolomide plus other chemotherapies had delayed recurrence, after at least 1 year (*P*-value = 0.007) compared with patients who received TZM alone. Around 86% of patients with non-*TP53* expressing tumors who received TZM plus other chemotherapies had recurrence after 1 year (*P*-value = 0.040)

*TMZ* Temozolomide

^a^Chi-Square Test; ^b^Fisher’s Exact Test; ^c^Mantel–Haenszel Chi-square test

#### Methylation analysis of the MGMT promotor in patients with different treatment modalities and recurrence intervals

A significant difference in recurrence interval was observed among glioblastoma patients with methylated and none-methylated *MGMT*, who received different chemotherapeutic protocols (adjusted *P*-value = 0.041). In total, 100% of patients with non-methylated *MGMT*, who received TMZ plus other chemotherapies, had recurrence after 1 year (*P*-value = 0.007) compared with those who received TMZ alone. However, this association was not significant in the *MGMT* methylation-positive subgroup (*P*-value = 0.667).

#### Recurrence interval analysis among glioblastoma patients with methylated and non-methylated MGMT after receiving different treatment modalities

**Fig. 2:**
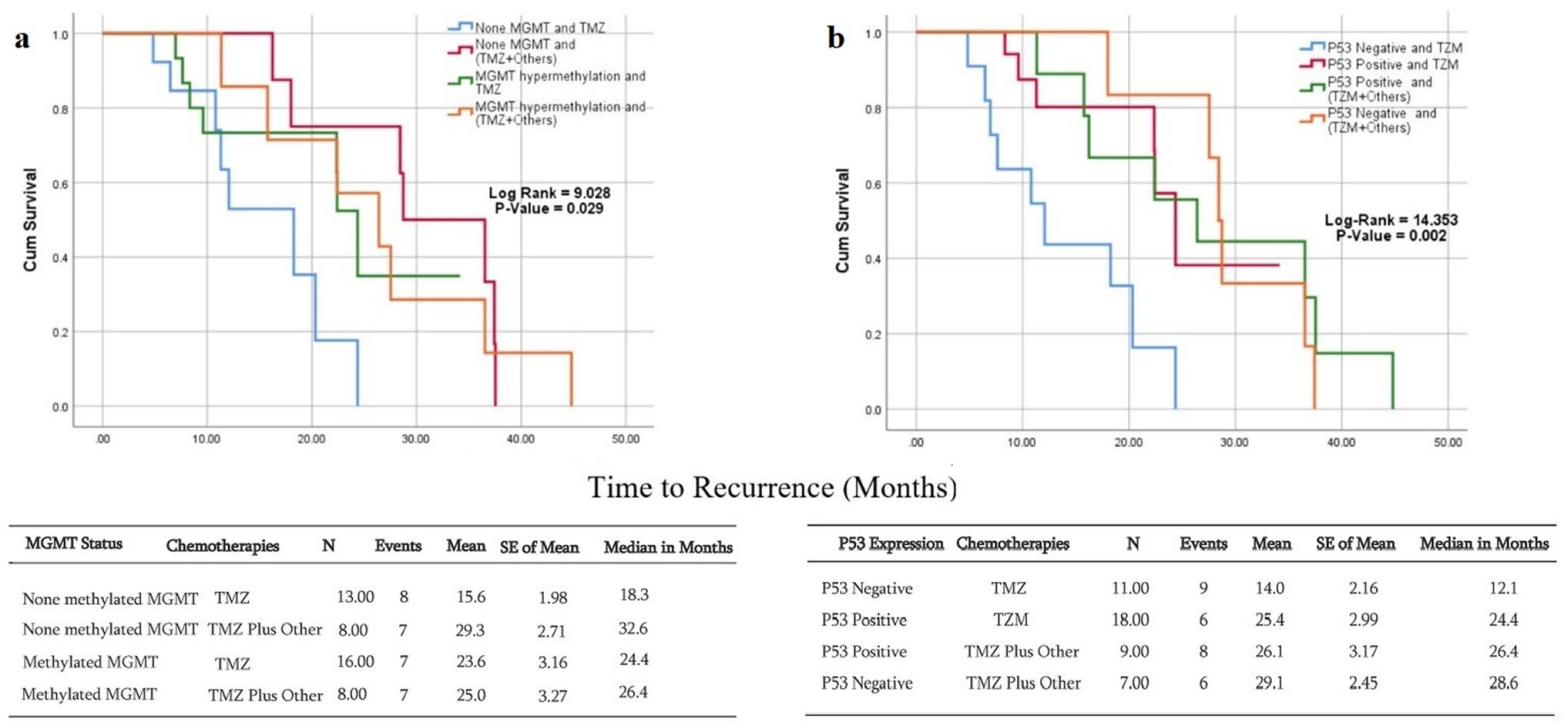
Kaplan–Meier analysis of the recurrence interval stratified for *MGMT*-methylation status and *TP53* expression. There was an overall significant difference in recurrence rate among glioblastoma patients receiving different chemotherapies, in regard to *MGMT* methylation status and *TP53* expression. The median recurrence rate for glioblastomas with non-methylated *MGMT* that received a regime of multiple chemotherapies was 32.6 months, while the median recurrence rate for glioblastomas with *TP53* expression that received a regime of multiple chemotherapies was 28.6 months

The median recurrence time for patients with non-methylated *MGMT*, who received TMZ plus additional chemotherapies, was 32.6 months. The median recurrence time for patients with non-methylated *MGMT* but who received TMZ alone was 18.3 months. This was not significantly different from the *MGMT* methylation-positive group who received either TMZ alone or TMZ with or without additional chemotherapies (median recurrence = 24.4–26.4 months) (Fig. 2a).

#### Recurrence interval analysis among glioblastoma patients with positive and negative TP53 expression status after receiving different treatment modalities

The median recurrence time for *TP53*-negative glioblastoma patients who received TMZ plus additional chemotherapies was 28.6 months. The median recurrence time for *TP53*-negative glioblastoma patients who received TMZ alone was 12.1 months. This was not significantly different from the of *TP53*-positive group who received either TMZ alone or TMZ with additional chemotherapies (median recurrence = 24.4–26.4 months) (Fig. 2b).

## Discussion

Glioblastoma is the most common primary brain tumor in adults. The standard treatment includes surgical resection, RT and chemotherapy, mostly with the alkylating agent, TMZ. Despite this approach, outcomes are usually poor [1]. While post-operative combined treatment is the primary treatment option for newly diagnosed glioblastoma, chemotherapy resistance is still common. The efficacy of chemotherapy as an adjunct to RT is controversial. Chemotherapy with individual treatments is rarely used to treat glioblastoma after surgery but occurs in patients who refuse radiotherapy and different treatment modalities.

Several parameters have been used to predict the prognosis of glioblastoma patients. Amongst these, methylation of the *MGMT* promoter is a biomarker for favorable outcome [2, 3]. *MGMT* is located on chromosome band 10q26. It is a DNA repair protein that rapidly reverses alkylation (including methylation) at the O6 position of guanine, thereby neutralizing the cytotoxic effects of alkylating agents, such as TMZ. A lack of *MGMT* repair capacity contributes to the genesis and progression of human cancers because it leads to the accumulation of DNA mutations and chromosomal instability [8]. The relative expression of *MGMT* can determine patient response to alkylating agents, and the epigenetic silencing of *MGMT* by promoter methylation plays an active role in regulating *MGMT* expression [7]. Therefore, elevated levels of *MGMT* activity, defined as non-methylated *MGMT*, in tumor tissue are associated with resistance to alkylating agents.

*MGMT* promoter methylation correlates with improved tumor progression-free survival in patients treated with TMZ [9, 10]. A meta-analysis of 34 clinical trials concluded that *MGMT* methylation was significantly associated with better overall survival in patients with glioblastoma [11]. Hegi et al. reported that the 18-month survival rate was 62% among patients with a methylated *MGMT* promoter compared with only 8% in the absence of promoter methylation [5]. Some studies also showed that *MGMT* promoter methylation was associated with improved overall survival in patients treated with RT and TMZ but not in patients initially treated with RT alone [8]. In our study, we found that RT with TMZ and additional chemotherapies (such as lomustine and/or procarbazine and bevacizumab), particularly in unmethylated *MGMT* tumors, was associated with a longer recurrence interval and prolonged survival rate. These observations have not been previously reported. Rapp et al. studied the impact of surgical resection, RT and concomitant TMZ in glioblastoma patients with methylated and non-methylated *MGMT*. They did not use *TP53* expression as a factor in the prognosis [12]; however, they concluded that tumor progression-free survival and overall survival rates are strongly determined by *MGMT* status.

Although the recurrence rate is lower and tumor progression-free survival is increased in glioblastoma patients with *MGMT* methylation compared with non-methylated glioblastoma, not all patients with *MGMT* methylation-positive glioblastoma respond to alkylating agents, and even those who respond eventually show relapse [5]. This might be because of a high rate of treatment resistance to alkylating agents in both groups. Wiewrodt et al. revealed that *MGMT* expression is related to a change in gene expression pattern that occurs during tumor growth and progression and that it might cause therapy-related resistance [13]. Strategies to overcome *MGMT*-mediated chemoresistance are being investigated. The use of MGMT inhibitors is limited by their hematological toxicity; therefore, another strategy has been to deplete MGMT activity in tumor tissue using a dose-dense temozolomide schedule. This has been used in non-methylated *MGMT* cases. Here, we found that the use of additional chemotherapies with TMZ treatment may have reduced the level of resistance and slowed the tumor recurrence rate to more than 1 year.

Mutations in the *TP53* gene have been frequently reported in glioblastoma [14, 15]. The immunohistochemical presence or absence of nuclear TP53 is highly significantly associated with the presence or absence of *TP53* variations. Significant correlation between *TP53* expression, *TP53* mutations, and *TP53* locus loss of heterozygosity has been shown [14, 16]. The regulation of *MGMT* involves TP53, with TP53 being required for induction and also down-regulation of its basal level of expression [4, 6]. In a recent population-based study of glioblastoma, a higher frequency of *TP53* mutations (G:C > A:T transition) was found in tumors with *MGMT* promoter methylation (25%) than in glioblastomas without *MGMT* methylation (16%) [17].

MGMT promotes methylation and *TP53* mutation or *P53* expression is insignificantly related. TP53 has an impact on the sensitivity of glioma cells to TMZ [6, 18]. Groenendijik et al. found no significant relationship between *TP53* mutation and *MGMT*-promotor methylation [19]. The *TP53* variant (H: C > A) is commonly found in *MGMT* methylated tumors while *TP53* was not mutated in seven cases [19]. In a study conducted by Wiewrodt et al., *TP53* expression was not significantly related to the level of *MGMT* expression, although a trend for lower *MGMT* activity in TP53-positive tumors was observed [20]. Zawlik et al. found a significantly higher frequency of *TP53* G:C > A:T transition mutations in glioblastomas with *MGMT* promoter methylation compared with glioblastomas without *MGMT* promoter methylation, whereas the total frequency of *TP53* mutations in glioblastoma with methylated and non-methylated *MGMT* promoters was similar [17]. Some in vitro studies have shown that lack of *TP53* expression can sensitize glioma cells to carmustine and TMZ [21, 22].

In our study, despite the *MGMT* promotor being methylated more frequently in tumors expressing *TP53*, no significant relationship was observed between *MGMT* and *TP53*. We also found that in glioblastomas where *TP53* was not expressed, treatment with TMZ and additional chemotherapeutic agents was associated with a low rate of tumor recurrence within 1 year, which was significantly different from the rate in patients treated with TMZ alone. This shows that lack of *TP53* expression may increase the sensitization of glioblastoma cells to additional chemotherapeutic agents and reduce the risk of TMZ resistance.

Finally, one limitation must be acknowledged in our study is, that the total number of cases analyzed for *MGMT* promoter methylation and *P53* expression is relatively low. Despite this limitation and to our best knowledge, this is the first study in Saudi Arabia that correlates these molecular biomarkers with recurrence-free interval in totally resected glioblastomas, reflecting the impact of adjuvant therapies as well as the specific type of chemotherapies on patient outcome. The results are in keeping with other conclusions from similar international studies.

## Conclusions

Our results show that TMZ with other chemotherapeutic agents, regardless the type and number of added drugs, produce a significantly better outcome (reduced recurrence interval) in glioblastoma without *TP53* expression or non-methylated *MGMT* promotor. This is because these glioblastomas have increased sensitivity to chemotherapies. Furthermore, *TP53* mutation or expression and *MGMT* methylation are significant independent prognostic factors. Further studies should be performed to understand the mechanisms of glioma cell sensitivity to different chemotherapeutic agents.

## Data Availability

The data that support the findings of this study are available from the corresponding author (MK) upon request.
